# Supplementary material for: Reconciling Mining with the Conservation of Cave Biodiversity: A Quantitative Baseline to Help Establish Conservation Priorities
Source: PLoS One. 2016 Dec 20;11(12):e0168348. doi: 10.1371/journal.pone.0168348 (PMC5173368; doi:10.1371/journal.pone.0168348)
Supplement: S1 Dataset — (ZIP) [file pone.0168348.s002.zip › Taxa/Serra Norte/SN_2013/N5S/Lista N5S-84.pdf]

CAVIDADE N5S-0084

Classe	Ordem	Fam/Outros	Gên/Outros	Espécie	S-U	U-S	Zon.
Arachnida	Acari	Cunaxidae		sp.1		X	E
Arachnida	Amblypygi	Phrynidae	*Heterophrynus*	*longicornis*	1	1	E
Arachnida	Araneae	Ochyroceratidae	*Ochyrocera*	sp.1	X		E
Arachnida	Araneae	Pholcidae	*Mesabolivar*	*aurantiacus*		X	E
Arachnida	Araneae	Salticidae		sp.2		X	E
Arachnida	Araneae	Theridiidae		jovem	X		E
Arachnida	Pseudoscorpiones	Chthoniidae		jovem		X	E
Chilopoda				jovem	1		E
Diplopoda	Polydesmida	Pyrgodesmidae		jovem	X		E
Entognatha	Collembola	Isotomidae		sp.1	X		E
Entognatha	Collembola	Sminthuroidea		sp.2	X		E
Insecta	Blattodea			jovem	1		E
Insecta	Coleoptera	Carabidae		sp.6		X	E
Insecta	Coleoptera	Dytiscidae		sp.4		X	E
Insecta	Diptera	Cecidomyiidae	Cecidomyiinae	sp.		X	E
Insecta	Diptera	Dolichopodidae		sp.	X		E
Insecta	Diptera	Phoridae		sp.		X	E
Insecta	Diptera	Psychodidae	Phlebotominae	sp.	X	X	E
Insecta	Heteroptera	Veliidae	*Paravelia*	sp.	X	X	E
Insecta	Homoptera	Cixiidae		sp.1	X	X	E
Insecta	Hymenoptera	Formicidae	*Gnamptogenys*	sp.1		X	E
Insecta	Hymenoptera	Formicidae	*Pachycondyla*	sp.2	X		E
Insecta	Hymenoptera	Formicidae	*Pheidole*	sp.3		X	E
Insecta	Isoptera	Termitidae	*Nasutitermes*	sp.		X	E
Insecta	Lepidoptera	Noctuoidea		sp.5		1	E
Insecta	Lepidoptera	Noctuoidea		sp.9	1		E
Insecta	Orthoptera	Phalangopsidae	*Paraclodes*	sp.	1	3	E
Insecta	Orthoptera	Phalangopsidae	*Phalangopsis*	sp.		4	E
Insecta	Psocoptera	Epipsocidae	*Epipsocus*	sp.1		X	E
Insecta	Psocoptera	Epipsocidae	*Epipsocus*	sp.2	X		E
Amphibia	Anura			sp.		1	E
Mammalia	Chiroptera	Furipteridae	*Furipterus*	*horrens*		1	E
Mammalia	Chiroptera	Phyllostomidae	*Trachops*	*cirrhosus*		1	E
